# Editorial Commentary for the Special Issue “Infectious Disease Prevention and Public Health Promotion”

**DOI:** 10.3390/healthcare11101422

**Published:** 2023-05-14

**Authors:** Vito Fiore

**Affiliations:** Unit of Infectious Diseases, Department of Medicine, Surgery and Pharmacy, University of Sassari, 07100 Sassari, Italy; vitofiore30010516@gmail.com

HIV testing and treatment, as well as sexually transmitted infections (STIs), remain popular topics for infectious disease specialists.

Regarding HIV, a good testing and treatment approach is becoming increasingly important to avoid delay in diagnosis, promote optimal infection control, and reduce infection transmission [1]. Furthermore, the lack of knowledge among the general population on HIV and STIs is still a barrier to overcome. The results of a web-based survey completed by 2183 participants revealed several gaps in general population awareness about HIV and STIs [2].

The recent human monkeypox outbreak represents a clear signal of the lack of knowledge on STIs, even among healthcare workers (HCWs). A survey conducted in Jordan, demonstrated many problems regarding the monkeypox outbreak, including conspiracy beliefs, the belief in the role of men who have sex with men in viral spread, as well as low diagnosis and management confidence levels among HCWs [3]. For these reasons, an improvement in knowledge, starting from surveys and questionnaires using social media, could represent an opportunity to reach the general population and develop tailored informational and educational campaigns.

Reducing the viral hepatitis as a major global public health threat represents another fundamental step, according to the WHO [4]. However, recent clusters of acute non HepA-E hepatitis among children aroused concern in the scientific community, particularly about its unknown etiology. Finally, the results reported by the ESCMID Study Group for Viral Hepatitis highlighted the impact of the group F human adenovirus serotype 41 (HAdV-F41), given its isolation in ~70% of cases [5]. This is an important finding, and physicians are recommended to conduct a genetic analysis of HAdV-F41 isolates to assess the potential changes in the viral genome and possibly alter virus behavior. In this case, knowledge is the first step towards effective treatment approach.

To date, COVID-19 remains a barrier for healthcare provision and use. The pandemic has severely damaged healthcare services, increased people’s anxiety and depression, and negatively influenced the progress in the approaches towards other infectious diseases. Even if mortality rates are lower owing to vaccines and early treatments, SARS-CoV-2 infection still spreads worldwide among both inpatients and outpatients.

There is a broad range of literature regarding COVID-19 treatment approaches (e.g., antiviral drugs and supplements) [6,7]. However, a knowledge gap remains regarding severely immunosuppressed patients. In fact, the most widely used antiviral drugs against SARS-CoV-2 infection have no immediate effect on the viral cycle’s threshold among immunosuppressed patients. Research should probably focus more on this subpopulation. Moreover, good predictors of its clinical evolution among inpatients are needed. In this field, complete blood cell count (CBC)-derived inflammatory indexes may represent a good strategy. There is increasing evidence regarding the relationship between CBC-derived ratios and patients’ outcome [8]. For this reason, CBC-derived indexes may help physicians to quickly differentiate inpatients with a higher risk of serious illness and death.

Antimicrobial resistance is one of the top 10 global public health threats facing humanity, according to the WHO. According to the ECDC, antimicrobial resistance in European Countries is increasing alarmingly [9]. The data regarding community-acquired infections pointed out the necessityof addressing the high burden of infectious diseases, particularly given the possibility of young adult carriers without symptoms [10]. Moreover, emerging infections with high rates of mortality, as well as the possibility of multidrug-resistant and pandrug-resistant strains, are rising, such as *Candida auris.* This fungal pathogen is difficult to control, survives for a long time in the environment, and colonizes patients for prolonged periods [11]. Therefore, antimicrobial stewardship has never been as important as in this historical moment.

In conclusion, tailored approaches and strategies for infectious disease prevention, treatment, and control should be increased to improve healthcare provision and use.
